# Autophagy-dependent cell cycle arrest in esophageal cancer cells exposed to dihydroartemisinin

**DOI:** 10.1186/s13020-020-00318-w

**Published:** 2020-04-25

**Authors:** Qiang Ma, Hebin Liao, Lei Xu, Qingrong Li, Jiang Zou, Ru Sun, Dan Xiao, Chang Liu, Wenjie Pu, Jibing Cheng, Xi Zhou, Guangcheng Huang, Lihua Yao, Xiaowu Zhong, Xiaolan Guo

**Affiliations:** 1 Department of Clinical Laboratory, Affiliated Hospital of North Sichuan Medical College, Nanchong, 637000 People’s Republic of China; 2 Translational Medicine Research Center, North Sichuan Medical College, Nanchong, 637000 People’s Republic of China; 3 Department of Laboratory Medicine, North Sichuan Medical College, Nanchong, 637000 People’s Republic of China; 4 Department of Blood Transfusion, Affiliated Hospital of North Sichuan Medical College, Nanchong, 637000 People’s Republic of China

**Keywords:** Cell cycle arrest, Autophagy, TRF2, Dihydroartemisinin, Esophageal cancer

## Abstract

**Background:**

Dihydroartemisinin (DHA), a derivate of artemisinin, is an effective antimalarial agent. DHA has been shown to exert anticancer activities to numerous cancer cells in the past few years, while the exact molecular mechanisms remain to be elucidated, especially in esophageal cancer.

**Methods:**

Crystal violet assay was conducted to determine the cell viability of human esophageal cancer cell line Eca109 treated with DHA. Tumor-bearing nude mice were employed to evaluate the anticancer effect of DHA in vivo. Soft agar and crystal violet assays were used to measure the tumorigenicity of Eca109 cells. Flow cytometry was performed to evaluate ROS or cell cycle distribution. GFP-LC3 plasmids were delivered into Eca109 cells to visualize autophagy induced by DHA under a fluorescence microscope. The mRNA and protein levels of each gene were tested by qRT-PCR and western blot, respectively.

**Results:**

Our results proved that DHA significantly reduced the viability of Eca109 cells in a dose- and time-dependent manner. Further investigation showed that DHA evidently induced cell cycle arrest at the G2/M phase in Eca109 cells. Mechanistically, DHA induced intracellular ROS generation and autophagy in Eca109 cells, while blocking ROS by an antioxidant NAC obviously inhibited autophagy. Furthermore, we found that telomere shelterin component TRF2 was down-regulated in Eca109 cells exposed to DHA through autophagy-dependent degradation, which could be rescued after autophagy was blocked by ROS inhibition. Moreover, the DNA damage response (DDR) was induced obviously in DHA treated cells. To further explore whether ROS or autophagy played a vital role in DHA induced cell cycle arrest, the cell cycle distribution of Eca109 cells was evaluated after ROS or autophagy blocking, and the results showed that autophagy, but not ROS, was essential for cell cycle arrest in DHA treated cells.

**Conclusion:**

Taken together, DHA showed anticancer effect on esophageal cancer cells through autophagy-dependent cell cycle arrest at the G2/M phase, which unveiled a novel mechanism of DHA as a chemotherapeutic agent, and the degradation of TRF2 followed by DDR might be responsible for this cell phenotype.

## Background

Esophageal cancer (EC) was diagnosed and identified as one of the leading causes of cancer death in China [1]. The risk factors of EC included genetic background, cigarette smoking, alcohol consumption, gastroesophageal reflux disease, diet, obesity and body composition [2]. EC is asymptomatic, most of the patients are at an advanced stage at initial diagnosis [3], consequently resulting in a poor prognosis [4]. Neoadjuvant therapy, surgery resection, radiotherapy, and chemotherapy are the main treatments for EC. Esophageal squamous cell cancer (ESCC), one of the most common histological subtype of EC in China [5], performed drug resistance during chemotherapy [6, 7]. For this reason, new chemotherapeutic agents or some “conventional drugs” own anticancer activities might be utilized for esophageal cancer treatment.

Dihydroartemisinin (DHA), an antimalarial drug derivative from artemisinin, was reported own powerful anticancer effect on numerous kinds of cancers in vitro and in vivo, such as prostate cancer [8], ovarian cancer [9, 10], cervical cancer [11], gallbladder cancer [12] and gastric cancer [13]. In esophageal cancer, Jiang and coauthors [14] reported that DHA performed a powerful anticancer effect toward EC cell lines Eca109 and Ec9706 in vitro and in vivo. Further study demonstrated that PKM2 was a key molecule involved in the anticancer effect of DHA on EC [15]. The anticancer mechanisms of DHA on esophageal cancer are known far from enough, further studies are urgently needed to elucidate the potential role of DHA as an anticancer agent.

Autophagy is an evolutionarily conserved eukaryotic process in maintaining intracellular homeostasis, which has a major role in protein and organelle degradation and metabolism [16]. It was also reported that autophagy was a double-edged sword and triggered the death of cancer cells under certain circumstances [17]. Established tumor cells require autophagy for cell growth and tumor promotion. For instance, downregulated EI24, an essential component of the autophagy pathway, significantly inhibited the proliferation of pancreatic tumor cells [18]. However, autophagy is suppressed in many human cancers. Allelic loss of the essential autophagy gene *beclin1* is frequent in human breast, ovarian, and prostate cancers [19]. Autophagic cell death is one of the major mechanisms that induced programmed cell death. It was found that autophagic cell death played an important role in anticancer drugs [20, 21]. DHA could induce autophagy in some human cancer cell lines, including esophageal cancer cells [22–24], while the precise mechanisms of DHA on cancer cells were still limited. In the present study, we explored the role of autophagy in DHA treated Eca109 cells and the associated mechanisms were identified as well.

## Materials and methods

### Reagents and antibodies

DMEM and FBS were purchased from Gibco (Grand Island, USA). Penicillin and Streptomycin were obtained from Solarbio (Beijing, China). Dihydroartemisinin (DHA) was purchased from Must Biotechnology (Chengdu, China). CQ and 3-MA were the products of Sigma-Aldrich (St. Louis, MO, USA). DMSO and DMF were purchased from Sigma-Aldrich (St. Louis, MO, USA) and used as solvents for DHA and NAC, respectively. NAC was purchased from Beyotime Biotechnology (Shanghai, China). The cell cycle detection kit was obtained from Keygen BioTECH (Nanjing, China). GFP-LC3 plasmids were a gift from Professor Yibin Deng at the University of Minnesota Hormel Institute. Lipofectamine 2000 reagent was provided by Invitrogen (Carlsbad, USA). The antibodies against P62, γ-H2AX, LC3, TRF2, GAPDH and goat anti-rabbit IgG were purchased from Cell Signaling Technology (Beverly, USA). The antibodies against CDK1, CyclinB1, and Cdc25c were kindly provided by HUABIO (Hangzhou, China). Goat anti-Rabbit IgG was purchased from BOSTER (Wuhan, China).

### Cell culture

Human esophageal squamous cell carcinoma (ESCC) cell line Eca109 was obtained from the translational medicine research center of North Sichuan Medical College. These ESCC cells were cultured in DMEM supplemented with 10% FBS at 37 °C in 5% CO_2_.

### Cell viability assay

Eca109 cells were seeded into a 6-well plate (Corning) at a density of 5 × 10^5^ cells per well in DMEM containing 10% FBS and incubated at 37 °C in 5% CO_2_. After 12 h, cells were treated with various concentrations of DHA for 48 h, or DHA at 100 μM for different time points, respectively. Cell viability was evaluated by crystal violet assay according to the literature [25]. Finally, the optical density of each well was measured at 590 nm (OD590) with a microplate reader.

### Tumor-bearing nude mice model construction and treatment

BALB/c male nude mice were purchased from the Beijing Laboratory Animal Research Center (Beijing, China). Animal care and experiments were performed with the approval of the animal ethical committee of North Sichuan Medical College. All animals were kept in a favorable environment and acclimated at 25 °C and 55% of humidity under natural light/dark conditions, with free access to a rodent diet and water. The experimental animals were acclimated for 1 week before the beginning of the study. The in vivo studies were conducted on 6-week-old male nude mice around 18–20 g. Eca109 cells were collected from cell culture by trypsinization and subcutaneously implanted (1 × 10^6^ cells in 100 µL of culture medium) in the upper-right flank of nude mice. When Eca109 xenograft volume reached approximately 100 mm^3^, the esophagus cancer mouse models were established successfully, and the nude mice were randomly divided into two groups. Mouse model were treated with DHA or DMSO by intraperitoneal injection every 2 days, and tumor length and width was measured by a vernier caliper until the 19th day when all the nude mice were sacrificed. The tumor volume was calculated by the formula: Volume (mm^3^) = Length × Width^2^/2.

### Plate colony formation assay

Plate colony formation assay was employed to measure the tumorigenicity of ESCC cells. Eca109 cells were seeded into a 6-well plate at a density of 2 × 10^3^ per well. 5 days later, the medium was renewed with 2 mL fresh serum-contained DMEM with DMSO or various concentrations of DHA. When cell clones that had formed from individual cells were directly observed by eyes, the medium was removed and cells were fixed with absolute methanol for 10 min and stained with 0.5% crystal violet solution for 10 min. Images were obtained from a scanner.

### Soft agar colony formation assay

Tumorigenicity of Eca109 cells was examined using published soft agar colony formation assay [26] with slight modification. Briefly, 3 mL of 3% agar solution in DMEM containing 10% FBS was added to a 6 cm plate. After 1 mL of 0.65% low melting agar solution was mixed with 1 mL of DMEM containing 10% FBS and 3 × 10^4^ cells, the mixture was added to the bottom layer of the 3% solidified agar. Then 1 mL DMEM containing 10% FBS was added into the plate and the medium was renewed every 3 days. After incubated for 9 days, the upper-medium was changed to DMEM containing 10% FBS and DHA (100μM). At last, the number of colony spheres was then counted and images were obtained from an optical microscope after 2 weeks.

### Cell cycle analysis

Eca109 cells were seeded into a 6-well plate at a density of 5 × 10^5^ per well and treated with or without 100μM DHA after 12 h. 48 h later, cells were collected and washed twice with ice-cold PBS, then fixed in 70% ethanol at 4 °C for 2 h. After fixing, cells were rehydrated with ice-cold PBS, stained with a DNA staining solution, and incubated for 30 min and tested using a FACSCalibur Flow Cytometry (BD Biosciences, CA, USA). Cell cycle profiles were analyzed by Modfit software (BD Biosciences).

### Intracellular ROS measurement

Eca109 cells were seeded into a 6-well plate at a density of 5 × 10^5^ per well and treated with or without 100μM DHA after 12 h. 48 h later, the medium was removed and cells were washed twice with ice-cold PBS. DCFH-DA was diluted by 1:1000 with DMEM without FBS, and the cells were then incubated with the diluted DCFH-DA at 37 °C. Half an hour later, the DCFH-DA was removed and cells were washed three times with ice-cold PBS. The fluorescence signal was observed by a fluorescence microscope and the mean fluorescence intensity was then measured for each group by FACSCalibur Flow Cytometry.

### RNA isolation and reverse transcription-polymerase chain reaction

Total RNA was extracted from cells treated with DMSO or DHA by the TRIzol reagent (Thermo Fisher Scientific, USA) following the manufacturer’s instructions. All the steps were operated in an RNase-free condition. Total RNA was dissolved in RNase-free water and the concentration was measured with micro-spectrophotometer Nanodrop 2000c (Thermo Fisher Scientific, USA). RNA concentration and purity were assessed according to the ratio of A260/A280 and A260/A230. The total RNA was reversed transcribed into cDNA by reverse transcription system kit (Roche life science, Switzerland).

### Fluorescence quantitative real-time PCR

cDNA was used as a template with the Roche qPCR Master Mix kit and the reaction was monitored in a Roche Lightcycler 96 to measure the expression level of *TRF2*. The housekeeping gene *β*-*actin* was used as a reference and their primers sequences were as follows: TRF2: sense: 5′-GACCTTCCAGCAGAAGATGCT-3′, antisense: 5′-GTTGGAGGATTCCGTAGCTG-3′, and the *β*-*actin* sense: 5′-GCAAGCAGGAGTATGACGAG-3′, antisense: 5′-CAAATAAAGCCATGCCAATC-3′. In brief, PCR (the total volume is 10μL) consisted of 2 × Master mix 5μL (Roche), forward primer 0.4μL (10 nM, Sangon, China), Reverse primer 0.4μL (10 nM, Sangon, China), cDNA 1μL and ddH_2_O 3.2μL. The thermal cycling for *TRF2* and *β*-*actin* PCR were initial denaturation at 95 °C for 10 min, the next step was 30 cycles of denaturation at 95 °C for 15 s followed by annealing at 62 °C for 30 s and extension at 72 °C for 20 s. The 2^-ΔΔCT^ method was used to calculate the relative expression of *TRF2*.

### GFP-LC3 plasmids transfection

Eca109 cells were seeded into a 6-well plate at a density of 5 × 10^5^ per well and transfected with GFP‑LC3 plasmids using Lipofectamine 2000 reagent after 24 h. The images were obtained by a fluorescence microscope from GFP‑LC3 plasmids transfected cells after treated with 100μM DHA for 24 h.

### Western blotting

Eca109 cells were treated with 100μM DHA or DMSO and lysed by RIPA buffer containing proteinase inhibitors. The protein samples were detected using SDS-PAGE (8–12% gel). Protein was quantified using a BCA assay. A total of 40 µg of protein was loaded into each lane of the SDS-PAGE gel and transferred onto a polyvinyl difluoride (PVDF) membrane. The PVDF Membranes were blocked in 5% BSA for 1 h at room temperature, and probed with specified primary antibodies at a dilution of 1:1000 overnight at 4 °C, followed by conjunction with horseradish peroxidase-conjugated secondary antibodies and incubated for 1 h at room temperature. Bands were visualized using an enhanced chemiluminescence kit (Merck KGaA) in vilber fusion FX7 spectra chemiluminescence apparatus (France).

### Crystal violet assay

Eca109 cells were washed three times with PBS at each destination of treatment and fixed in absolute methyl alcohol for 10 min. Cells were stained in 0.1% crystal violet for 30 min and washed three times with ddH_2_O. The images were obtained by a scanner. Subsequently, the crystal violet was dissolved by 10% acetic acid and the OD values at 590 nm were obtained from a microplate reader.

### Statistical analysis

All in vitro experiments were repeated at least three times. Student’s *t* test or one-way analysis of variance (ANOVA) was used to compare differences between two groups or among multiple groups with SPSS 19.0 statistics software, respectively. Differences with *P* < 0.05 were considered statistically significant.

## Results

### DHA inhibits the proliferation ability of Eca109 cells in vitro and in vivo

**Fig. 1:**
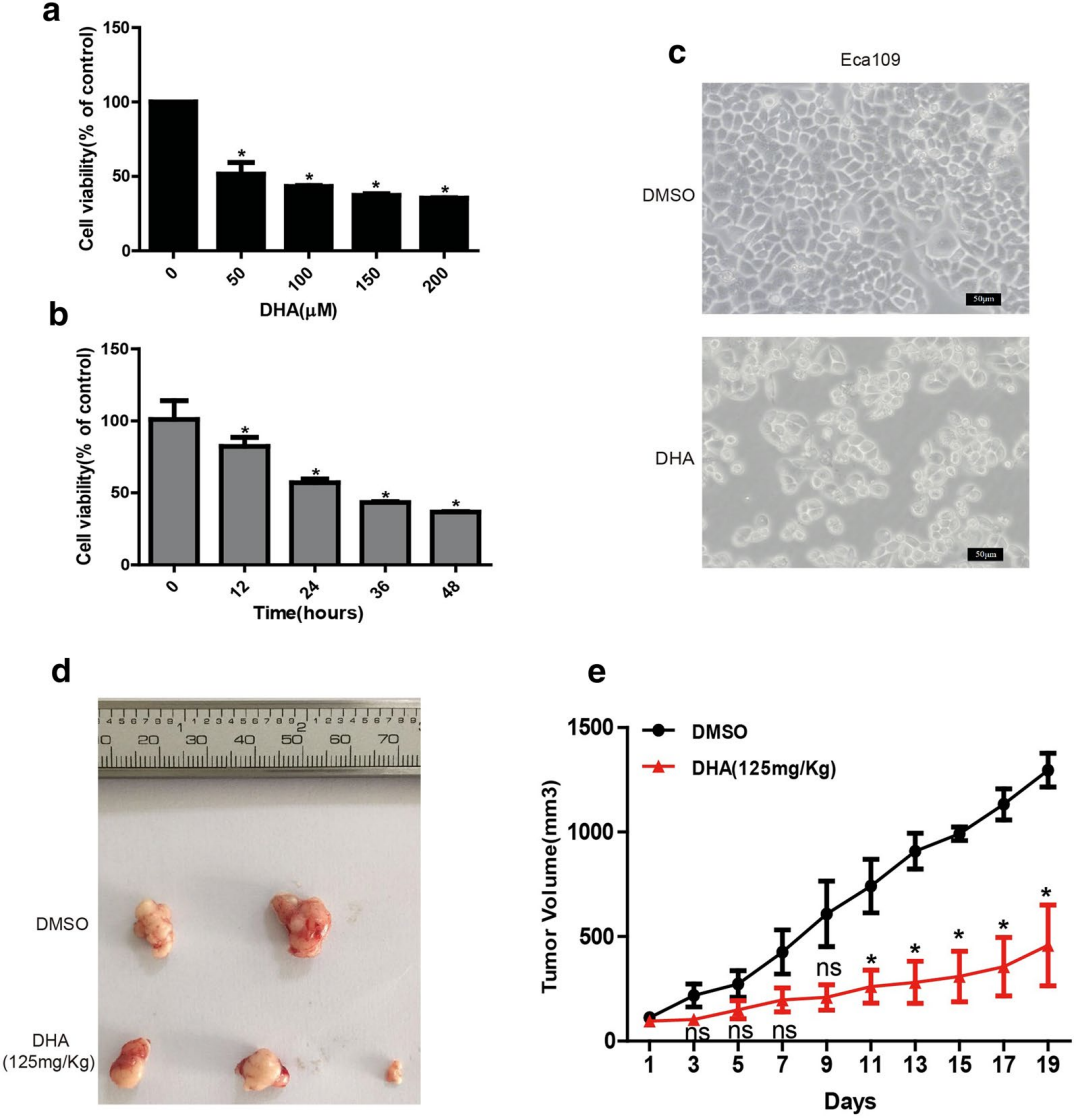
The anticancer effect of DHA on Eca109 cells in vitro and in vivo. **a**, **b** Cells were incubated with 0, 50, 100, 150, 200 μM of DHA for 48 h (**a**) or treated with 100 μM of DHA for 0, 12, 24, 36, 48 h (**b**) and then were collected for measuring the percentage of viable cells with crystal violet assay. (**c**) The morphological characteristics of Eca109 cells after treated with DHA (100 μM) or DMSO. (**d**, **e**) The tumor size (**d**) and proliferation curve (**e**) from esophageal cancer xenograft from mice. Data represent mean ± S.D. *p < 0.05 was significant difference between DHA-treated and control groups

To study the toxic effect of DHA on esophageal cancer cells in vitro, crystal violet assay was performed to evaluate the viability of Eca109 cells after treated with different concentrations of DHA (0, 50, 100, 150 and 200 μM) for 48 h. The results showed that DHA inhibited the viability of Eca109 cells in a dose-dependent manner (Fig. 1a), and the IC50 of DHA at 48 h was approximately 100 μM. We further treated Eca109 cells with 100 μM DHA at different time points (0, 12, 24, 36, 48 h) and found that the viable cell number decreased significantly with the increase of DHA treatment duration (Fig. 1b). Furthermore, cell morphology was observed through an optical microscope after exposed to 100 μM DHA for 48 h. The results showed that Eca109 cells had significant morphological alteration after DHA treatment compared with DMSO, showing smoother and smaller, disconnected from dishes gradually (Fig. 1c). Moreover, we constructed the tumor-bearing nude mice model with Eca109 cells to evaluate the anticancer effect of DHA on esophageal cancer in vivo. Models were treated with DHA at 125 mg per kilogram body weight by intraperitoneal injection every 2 days, and the width and length of tumors were measured before each treatment. We found that DHA could inhibit esophageal cancer cells growth in vivo effectively (Fig. 1d, e).

### DHA restrains tumorigenicity of Eca109 cells and induces cell cycle arrest

**Fig. 2:**
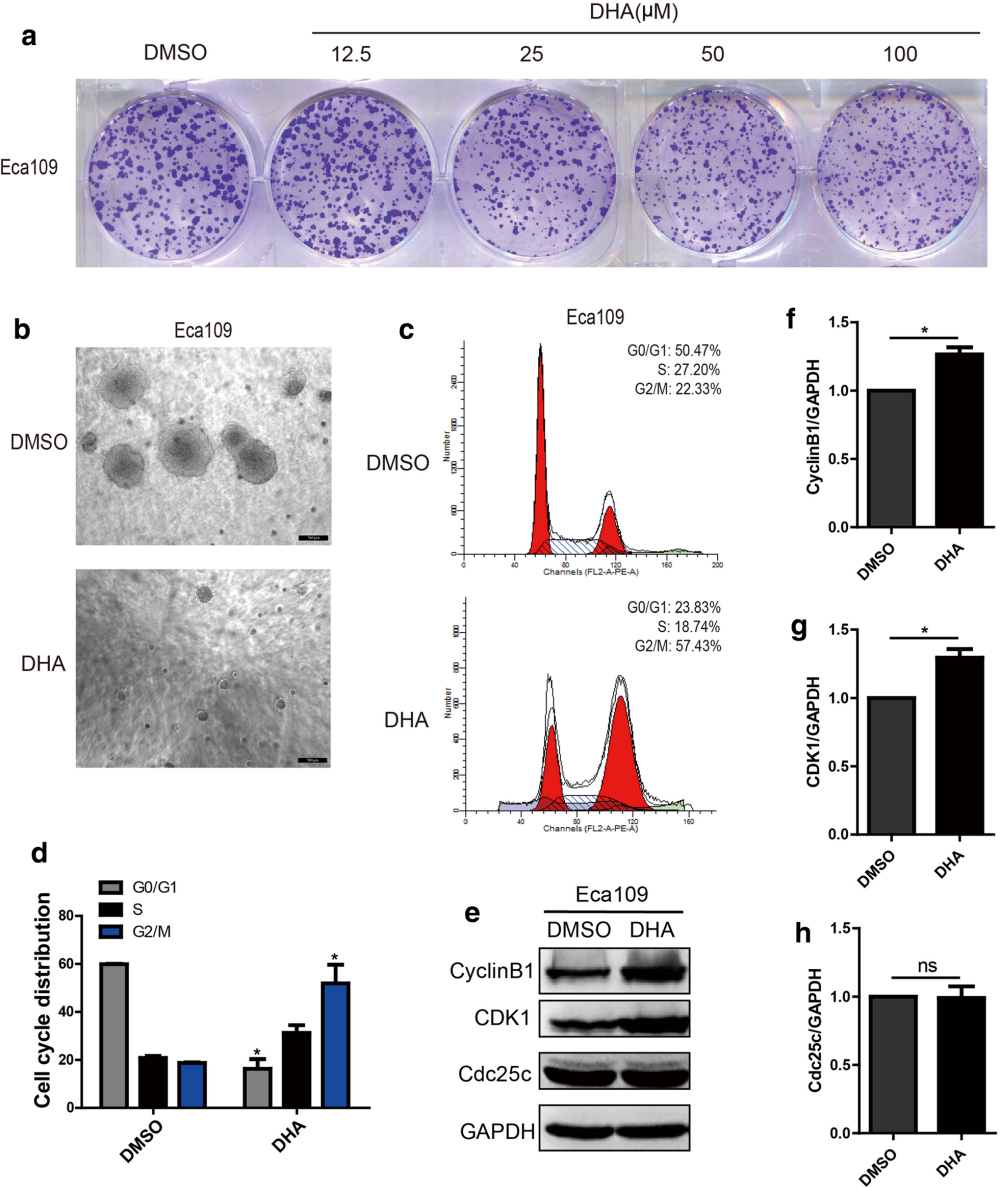
DHA inhibits the tumorigenicity of Eca109 cells and induces cell cycle arrest. **a**, **b** Represent images of plate colony formation assay (**a**) and soft agar assay (**b**) from Eca109 cells treated with DHA (100 μM) or DMSO. (**c**, **d**) Cell cycle distribution of Eca109 cells after treated with DHA (100 μM) or DMSO (**c**) and the statistical chart (**d**). (**e**–**h**) The expression of cell cycle-related proteins in DHA (100 μM) treated Eca109 cells (**e**) and the statistical charts (**f–h**). Data represent mean ± S.D. *p < 0.05 was significant difference between DHA-treated and control groups

To further explore whether DHA treatment could affect tumorigenicity of Eca109, plate colony formation assay and soft agar assay were employed after cells were treated with DHA or DMSO. As shown in Fig. 2a, the colony spheres of Eca109 cells were less or smaller after treated with 100 μM DHA compared with that treated with DMSO. The results were consistent with those findings from the soft agar assay (Fig. 2b). Moreover, we further investigated whether DHA contributed to the cell cycle distribution of Eca109 cells. Eca109 cells were treated with DHA at 100 μM for 48 h and the cell cycle distribution was evaluated by PI staining. As shown in Fig. 2c, d, the cell cycle distribution of the G2/M phase markedly increased in Eca109 cells treated with DHA. The molecular mechanisms showed that the up-regulation of cyclinB1 and CDK1, but not Cdc25c, might be involved in DHA induced cell cycle arrest at the G2/M phase (Fig. 2e–h).

### DHA induces intracellular ROS generation in Eca109 cells

**Fig. 3:**
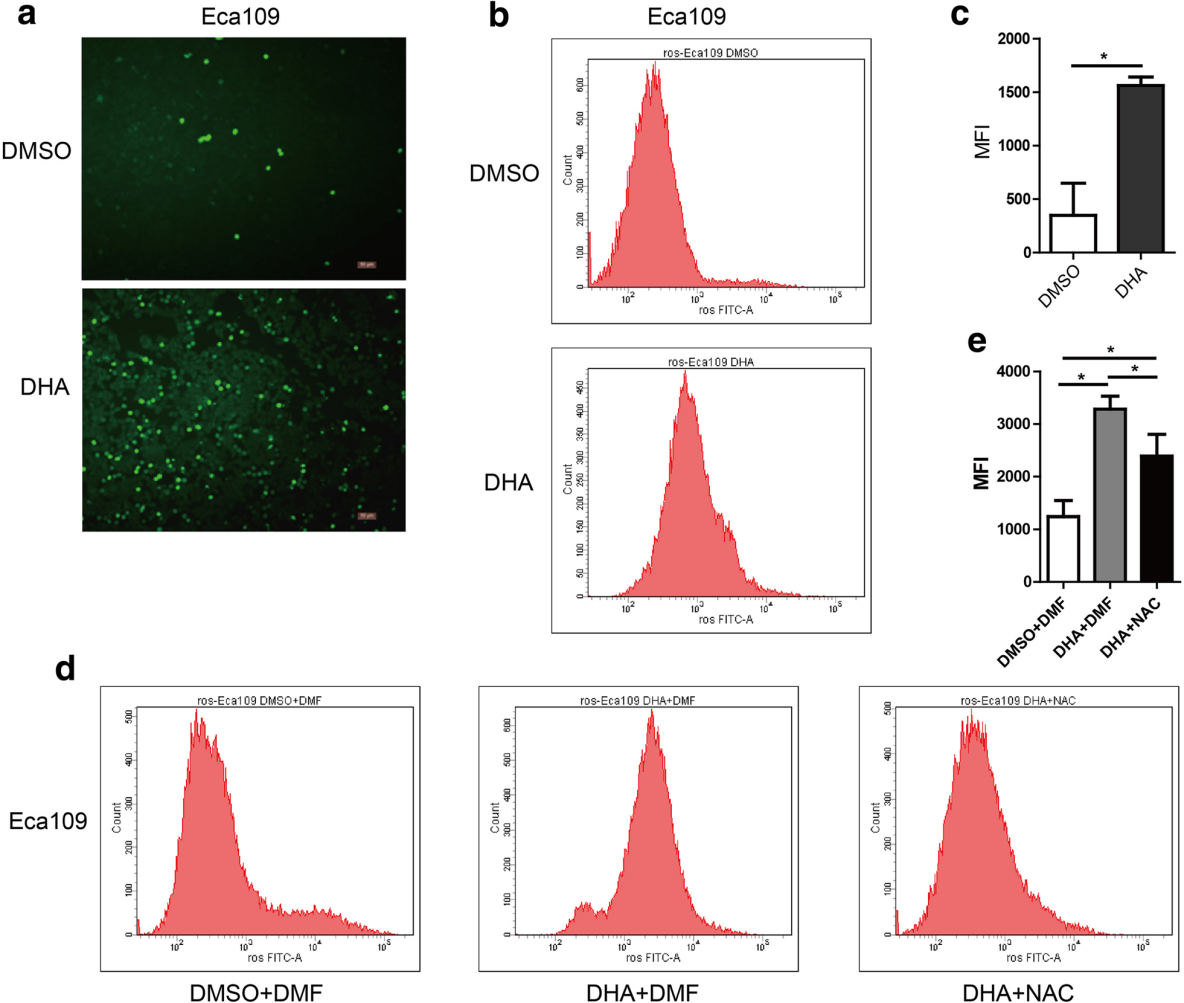
DHA induces intracellular ROS generation of Eca109 cells after treated with DHA. **a** Images from a fluorescence microscope after cells treated with DHA followed by DCFH-DA co-incubation. (**b**, **c**) The mean fluorescence intensity (MFI) of cells treated with DHA (100 μM) (**b**) and the statistical chart (**c**). (**d, e**) The change of MFI in cells treated with DHA (100 μM) plus NAC (5 mM) (**d**) and the statistical chart (**e**). Data represent mean ± S.D. *p < 0.05 was significant difference between DHA-treated and control groups

As most researchers found, the endoperoxide function of DHA might associate with its anticancer activity. Previous studies showed that DHA induced intracellular reactive oxygen species (ROS) generation in glioma cells and ovarian cancer cells [9, 27], whether DHA could also induce ROS generation in esophageal cancer cells remains unknown. We treated Eca109 cells with DHA at the concentration of 100 μM for 48 h and incubated cells with DCFH-DA for 30 min. Next, a fluorescence microscope and flow cytometry were employed to observe the green fluorescence signal and measure the mean fluorescence intensity (MFI) of Eca109 cells, respectively. It was shown that the MFI in Eca109 cells treated with DHA was higher than that with DMSO (Fig. 3a–c), and ROS induced by DHA could be repressed by NAC (Fig. 3d, e).

### DHA induces autophagy in Eca109 cells

**Fig. 4:**
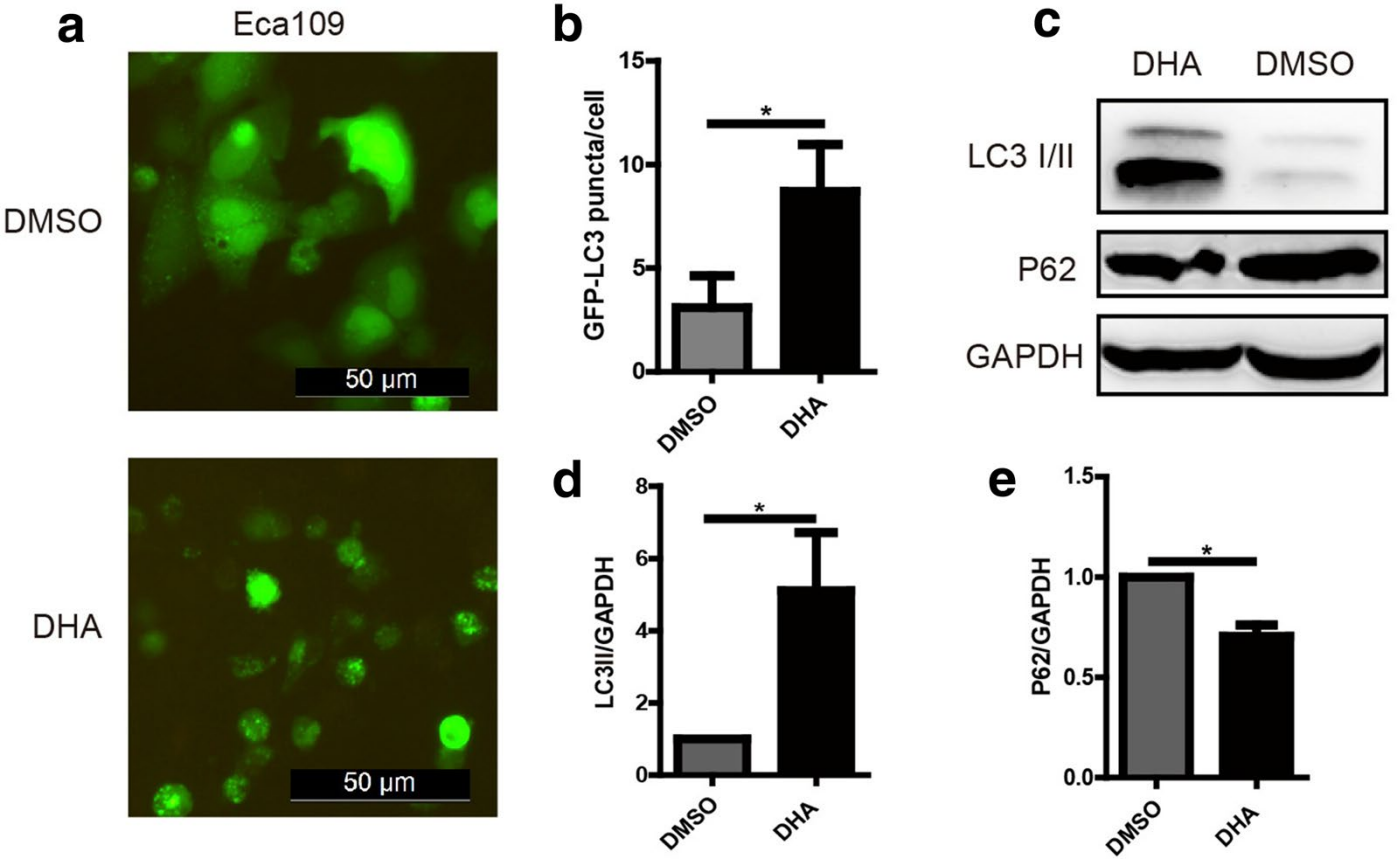
DHA induces autophagy in Eca109 cells. **a**, **b** GFP-LC3 puncta formation in DHA (100 μM) treated cells (**a**) and the statistical chart (**b**). **c**–**e** The expression of LC3 and P62 protein in cells treated with DHA (100 μM) or DMSO (C) and the statistical charts (**d**, **e**). Data represent mean ± S.D. *p < 0.05 was significant difference between DHA-treated and control group

Studies indicated that DHA induced autophagy in different types of cancer cells [22, 23, 28], whether DHA could mediate autophagy in esophageal cancer remains to be clarified. We transfected GFP-LC3 plasmids into Eca109 cells with the help of lipofectamine 2000 reagent, and massive green LC3 puncta could be observed post 48 h in cells treated with DHA compared with DMSO (Fig. 4a, b), which indicated that DHA could initiate autophagy in Eca109 cells. Moreover, the expression of LC3 and P62 were detected in Eca109 cells treated with DHA or DMSO. The results showed that LC3IIwas significantly upregulated while P62 was evidently downregulated in DHA treated cells (Fig. 4c–e), which revealed that DHA could induce autophagy in Eca109 cells.

### DHA degrades telomere shelterin component TRF2 through autophagy

**Fig. 5:**
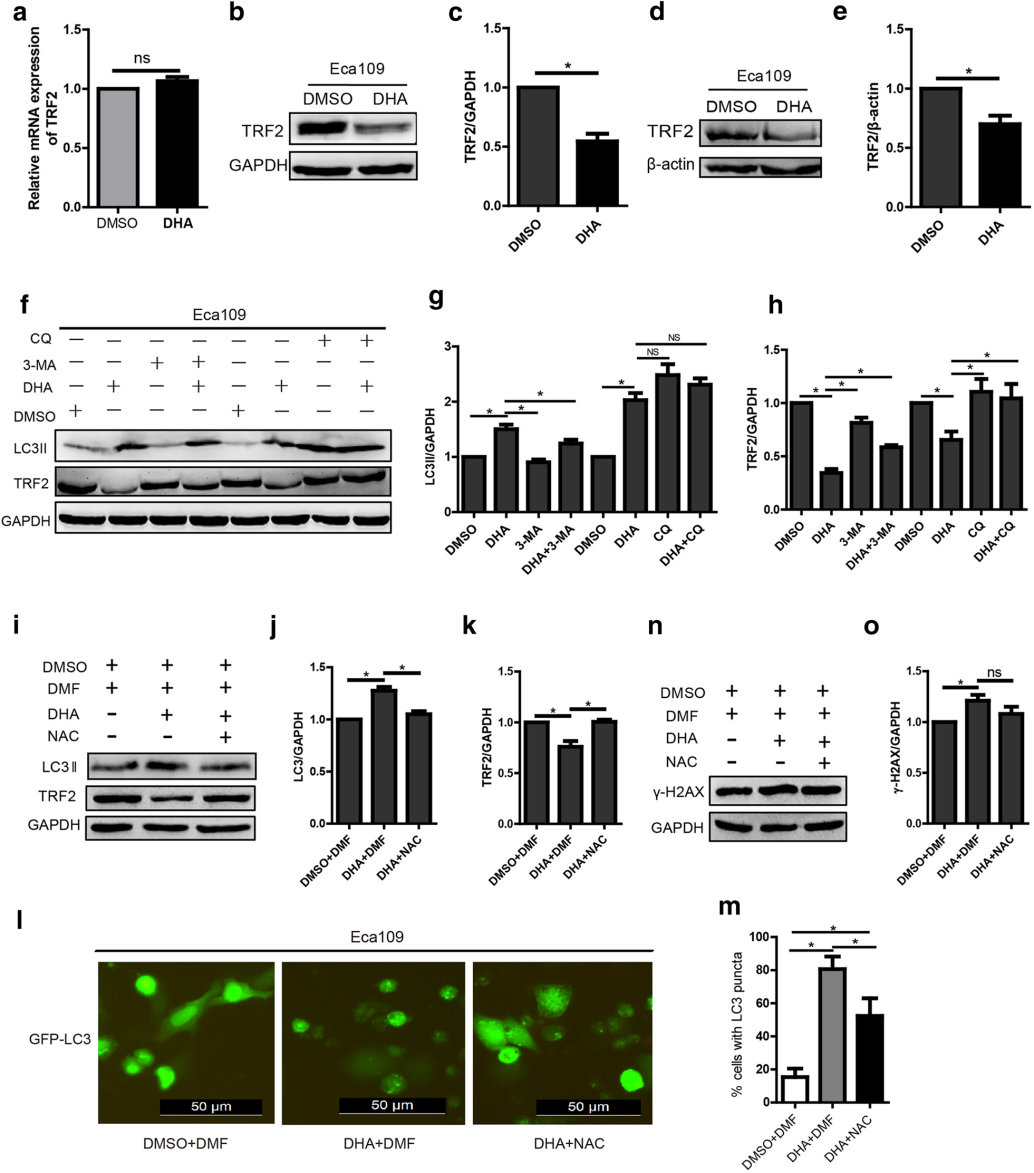
DHA downregulates the expression of TRF2 and the mechanisms. **a**–**c** The mRNA (**a**) and protein (**b**) expression of TRF2 in cells treated with DHA (100 μM) and the statistical chart (**c**). **d**, **e** The expression of TRF2 protein in tumor tissues from the xenograft mouse model treated with DHA (100 μM) or DMSO (**d**) and the statistical chart (**e**). **f**–**h** The expression of LC-3 and TRF2 in DHA (100 μM) treated cells after autophagy was inhibited by 3-MA (10 mM) or CQ (20 μM) (**f**) and the statistical chart (**g**, **h**). (**i**–**k**) The expression of LC-3 and TRF2 in DHA (100 μM) treated cells after ROS was blocked by NAC (5 mM) (**i**) and the statistical chart (**j**, **k**). **l**, **m** GFP-LC3 expression and puncta formation in DHA (100 μM) treated cells (**l**) and the statistical chart (**m**). **n**, **o** The expression of γ-H2AX in DHA (100 μM) treated cells after ROS was blocked by NAC (5 mM) (**n**) and the statistical chart (**o**). Data represent mean ± S.D. *p < 0.05 was significant difference between DHA-treated and control group

Previous study showed that gradual loss of TRF2 promoted a DNA damage response in U2OS cells [29], our previous study showed that DHA could downregulate telomere shelterin component TPP1 in hepatocellular carcinoma [30]. Whether DHA could also induce dysregulation of telomere shelterin in esophageal cancer cells remains unknown. In the present study, qRT-PCR and western blot were employed to measure the expression of TRF2 in RNA and protein levels, respectively. We found that the protein level, but not the RNA level, of TRF2 in DHA treated Eca109 cells was markedly reduced, which indicated that DHA regulated the expression of TRF2 post-transcriptionally (Fig. 5a–e). As we know, the autophagy-lysosome pathway is one of the main pathways of protein degradation. As abovementioned that DHA could induce autophagy in Eca109 cells, we then utilized 3-MA or CQ to inhibit autophagy and measured the expression of TRF2. We found that TRF2 was evidently increased in cells treated with CQ plus DHA or 3-MA plus DHA, compared with cells treated with DHA only, which demonstrated that TRF2 degradation induced by DHA was autophagy-dependent (Fig. 5f–h).

### Blockage of ROS induced by DHA could attenuate autophagy and partially reverse TRF2 degradation in Eca109 cells

ROS and autophagy are two key factors regulating cellular homeostasis in the human body. Data showed that ROS could involve in the process of autophagy and vice versa [31]. Here, the relationship between ROS and autophagy induced by DHA is needed to be clarified. As shown in Fig. 5i–m, NAC, an antioxidant, could inhibit the upregulation or puncta formation of LC3 induced by DHA. Moreover, NAC could also inhibit the downregulation of TRF2 induced by DHA. These results demonstrated that the degradation of TRF2 by autophagy was induced by ROS in DHA treated Eca109 cells. Furthermore, we found that DHA could upregulate the expression of γ-H2AX and inhibit by NAC, which indicated that DHA could trigger the DNA damage response (DDR) in Eca109 cells (Fig. 5n, o).

### Inhibition of autophagy partially reverse cell cycle arrest in Eca109 cells treated with DHA

**Fig. 6:**
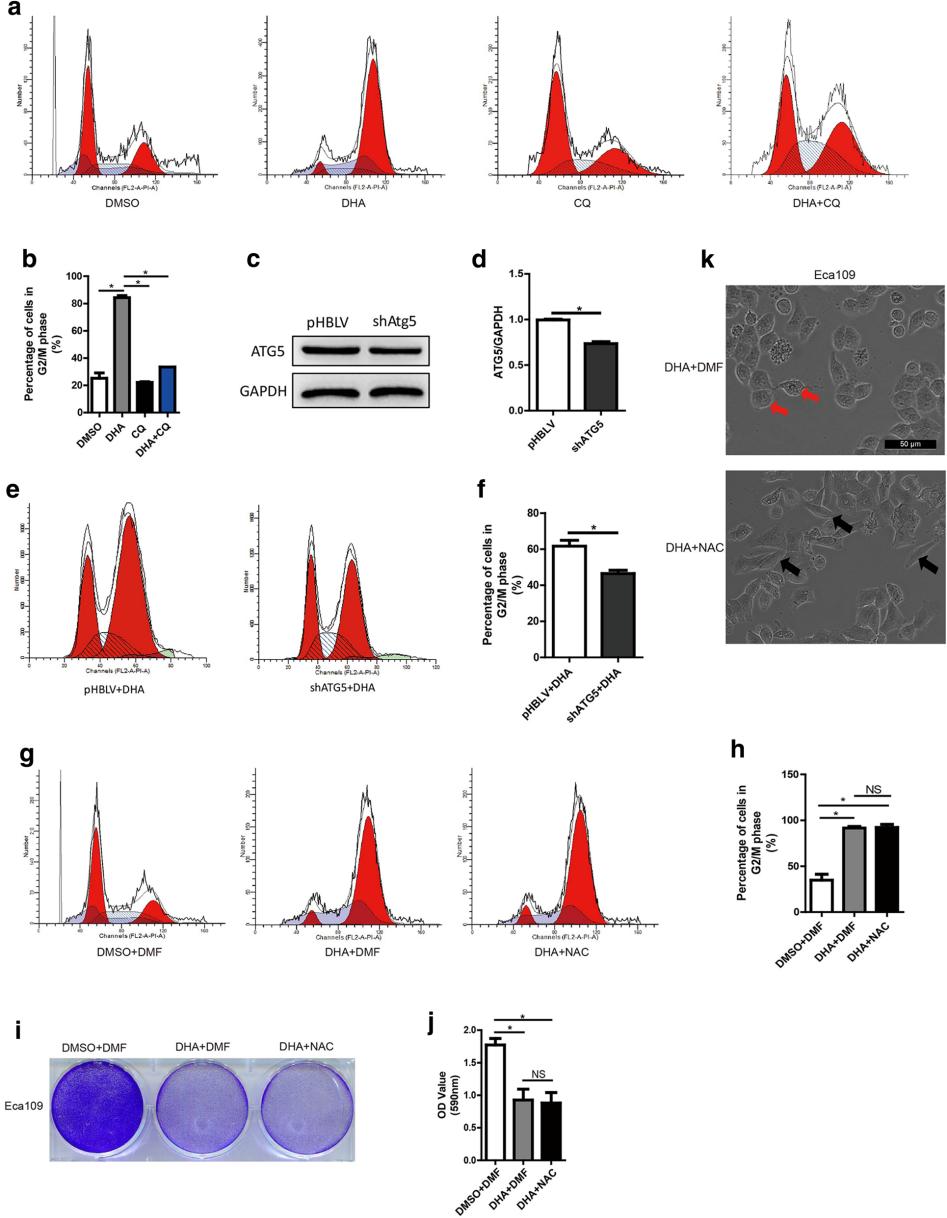
The evaluation of ROS and autophagy on cell cycle distribution in DHA treated cells. (**a**, **b**) Cell cycle distribution of cells treated with DHA (100 μM) and/or CQ (20 μM) (**a**) and the statistical chart (**b**). (**c**, **d**) The expression of ATG5 in cells transfected with *shAtg5* (**c**) and the statistical chart (**d**). (**e**, **f**) Cell cycle distribution of cells treated with DHA (100 μM) after *ATG5* was knockdown (**e**) and the statistical chart (**f**). (**g**, **h**) Cell cycle distribution of cells treated with DHA and/or NAC (5 mM) (**g**) and the statistical chart (**h**). (**i**, **j**) The effect of ROS on cell viability in DHA treated cells (**i**) and the statistical chart (**j**). (**k**) The effect of ROS on cell morphology in DHA treated cells

From the results mentioned above, we know that DHA could degrade TRF2 via autophagy induced by ROS, followed by inducing DDR in Eca109 cells. Whether ROS or autophagy played a vital role in DHA treated Eca109 cells remains unknown. As illustrated in Fig. 6a, b, compared with Eca109 cells treated with DHA only, the percentage of cells in the G2/M phase decreased significantly in cells treated with DHA combined with CQ, which indicated that autophagy played a key role in DHA treated Eca109 cells. Moreover, *ATG5* knockdown were also applied to confirm the potential role of autophagy in DHA induced cell cycle arrest in Eca109 cells, which showed a similar outcome as CQ did (Fig. 6c–f). Furthermore, we found that the difference of cells at the G2/M phase was not significant between cells treated with DHA plus DMF and DHA plus NAC (Fig. 6g, h). Crystal violet assay showed that inhibition of ROS by NAC had no obvious effect on the viability of Eca109 cells (Fig. 6i, j). The cellular morphology observed by an optical microscope showed that inhibition of ROS by NAC in cells treated with DHA decreased the percentage of smoother cells (Fig. 6k), which revealed that NAC could attenuate the toxic effect of DHA on Eca109 cells. Taken together, these results demonstrated that autophagy, but not ROS, played a vital role in DHA induced cell cycle arrest at the G2/M phase.

## Discussion

As an effective antimalarials reagent, artemisinin and its derivatives saved millions of people’s life. Youyou Tu awarded the Nobel Prize in physiology or medicine in 2015 for her great contribution to discover the antimalarial effect of artemisinin. Dihydroartemisinin (DHA), one of the derivatives of artemisinin, was found to own anticancer effects on numerous kinds of cancers [9, 10, 32]. The study of the anticancer effect of DHA on esophageal cancer is limited. In the present study, we found that DHA significantly inhibited Eca109 cell proliferation in vitro and in vivo, which was consistent with previous studies [24]. After treated with DHA, part of Eca109 cells became smoother and disconnected with the dish gradually. This phenotype of esophageal cancer cells treated with DHA was similar to other cancer cells treated with DHA [22]. Moreover, we found that DHA could inhibit the tumorigenicity of Eca109 cells in vitro evaluated by plate colony formation assay and soft agar assay. Furthermore, DHA could also induce cell cycle arrest at the G2/M phase in Eca109 cells by upregulating the expression of cyclinB1 and CDK1.

DHA possesses an unusual intramolecular endoperoxide bridge, which can be activated by heme or ferrous iron to produce the cytotoxic reactive oxygen species (ROS) [33]. For further evaluation whether ROS increased in DHA treated esophageal cancer cells, we measured the intracellular ROS level of Eca109 after treated with DHA. It was shown that DHA significantly increased the intracellular ROS level of Eca109 cells, especially in those who became smoother and smaller after treated with DHA. The ROS induced by DHA in Eca109 cells could be attenuated by antioxidant NAC. ROS refers to a group of reactive oxidant molecules and free radicals derived from molecular oxygen. Excessive ROS in cells caused double-stranded DNA breaks and induced cell cycle redistribution [34]. Moreover, ROS could initiate autophagosome formation and autophagic degradation acting as cellular signaling molecules [35]. In the current study, we found that DHA significantly induced autophagy in Eca109 cells. After ROS was inhibited by NAC in DHA treated Eca109 cells, the expression of LC3 was significantly downregulated, which demonstrated that autophagy induced by DHA was ROS-dependent.

Telomere is a cap structure located at the end of each chromosome in eukaryotic cells. It consists of telomere repetitive nucleotide sequences (TTAGGG) and shelterin. The shelterin complex is a key protein component and consists of six polypeptides: TRF1, TRF2, RAP1, TIN2, TPP1 and POT1 [36]. Among these components, TRF2 plays a vital role in maintaining telomere integrity [37]. A previous study showed that some synthetic compounds could inhibit the expression of TRF2 by binding with its DNA binding site causing blockage of its interaction with telomeric DNA [38]. Whether DHA could also inhibit the expression of TRF2 is unknown. We treated Eca109 cells with DHA and evaluated the expression of TRF2 on mRNA and protein levels. It was shown that DHA downregulated TRF2 on the protein level rather than the mRNA level, which was verified in vivo as well. As we know that the autophagy-lysosome pathway is one of the main pathways for protein degradation. From our present results, we inferred that DHA induced autophagy in Eca109 cells might contribute to TRF2 degradation. We then inhibited autophagy by CQ or 3-MA before cells treated with DHA and found that the expression of TRF2 was rescued after autophagy was blocked. These results demonstrated that autophagy played a vital role in TRF2 degradation by DHA. Given the crucial role of TRF2 in telomere integrity maintenance, the downregulation of TRF2 could further cause the DNA damage response (DDR). We measured the DDR marker γ-H2AX in DHA treated Eca109 cells and found that DHA significantly upregulated the expression of γ-H2AX. These results indicated that TRF2 degraded by DHA in Eca109 cells caused DDR. A previous study showed that ROS induced by polyhexamethylene guanidine phosphate caused DDR in lung epithelial cells [39]. In our study, we found that inhibited ROS by NAC could not significantly reduce the expression of γ-H2AX, which indicated that DDR induced by DHA might be partially through ROS, the exact mechanisms should be uncovered further.

From the results above we know that ROS and autophagy are two key events associated with the anticancer effect of DHA on Eca109 cells. Whether ROS and autophagy are correlated with cell cycle arrest is not clear. We inhibited ROS by NAC to evaluate the distribution of cell cycle of Eca109 cells treated with DHA. We found that ROS could not affect the viability or cell cycle distribution of Eca109 cells treated with DHA. However, we could see that the number of smoother cells in Eca109 cells treated with DHA plus NAC decreased compared with cells treated with DHA only. These results indicated that reduced ROS could attenuate the toxic effect of DHA on Eca109 cells, although it was not strong enough to reduce the killing effect of DHA within a limited administration time. We then inhibited autophagy in Eca109 cells by CQ and *ATG5* knockdown before DHA treatment and found that the percentage of G2/M phase in cells treated with CQ plus DHA or sh*ATG5* plus DHA was significantly decreased compared with that treated with DHA only, respectively. These results prove that autophagy is a key event during DHA induced cell cycle arrest at the G2/M phase in Eca109 cells.

In the present study, we demonstrated that DHA induces Eca109 cells G2/M phase arrest via the ROS-autophagy-TRF2-DDR pathway and clarified a new anticancer mechanism for DHA. We elucidated the role of ROS and autophagy in cell viability and cell cycle arrest, while failed to uncover the exact role of TRF2 in DHA treated cells because of the absence of overexpressing plasmids for TRF2. Taken together, our findings reveal a novel anticancer molecular mechanism of DHA, which provides more potential for DHA as an anticancer drug candidate in the future.

## Conclusion

The results from the present study suggest that DHA has potent anti-tumor effects on esophageal cancer, which might be due to the induction of ROS-mediated autophagy and TRF2 degradation, and cell cycle arrest at the G2/M phase. These findings provide scientific evidence supporting the potential use of DHA as an anticancer agent candidate for esophageal cancer.

## Data Availability

The datasets used and/or analyzed during the current study are available from the corresponding author on reasonable request.

## Abbreviations

DHA: Dihydroartemisinin
ROS: Reactive oxygen Species
NAC: N-acetyl-l-cysteine
DDR: DNA Damage response
TRF2: Telomere repeat binding factor-2
EC: Esophageal cancer
ESCC: Esophageal squamous cell carcinoma
DMEM: Dulbecco’s modified eagle medium
FBS: Fetal bovine serum
DMSO: Dimethylsulfoxide
DMF: Dimethyl formamide
DCFH-DA: 2′,7′-Dichlorodihydrofluorescein diacetate
TRIzol: Total RNA isolation reagent
PVDF: Polyvinylidene fluoride
ANOVA: Analysis of variance
IC50: Half maximal inhibitory concentration
qRT-PCR: Quantitative real-time polymerase chain reaction
3-MA: 3-Methyladenine
CQ: Chloroquine
